# Protocol: Prospective observational study investigating the prevalence and clinical outcome of portopulmonary hypertension in Japanese patients with chronic liver disease

**DOI:** 10.1371/journal.pone.0249435

**Published:** 2021-04-01

**Authors:** Shun-ichi Wakabayashi, Satoru Joshita, Kazuhiro Kimura, Hirohiko Motoki, Hiroyuki Kobayashi, Yuki Yamashita, Ayumi Sugiura, Tomoo Yamazaki, Koichiro Kuwahara, Takeji Umemura

**Affiliations:** 1 Department of Medicine, Division of Gastroenterology and Hepatology, Shinshu University School of Medicine, Matsumoto, Japan; 2 Department of Cardiovascular Medicine, Shinshu University School of Medicine, Matsumoto, Japan; 3 Department of Life Innovation, Institute for Biomedical Sciences, Shinshu University, Matsumoto, Japan; Nihon University School of Medicine, JAPAN

## Abstract

**Background:**

Portopulmonary hypertension (PoPH) is a subtype of the pulmonary arterial hypertension (PAH) associated with portal hypertension. There is a dissociation between the proportion of PoPH in PAH and that of PoPH in patients with liver cirrhosis, suggesting PoPH underdiagnosis and an incomplete understanding of this entity in the clinical setting. Specifically, real-world data in Japan is largely unknown as compared with in Europe and the United States. The present study aims to elucidate the prevalence and etiology of PoPH in Japanese patients with chronic liver disease.

**Methods and design:**

In this prospective, single-center, observational investigation of PoPH patients with chronic liver disease, a targeted 2,500 Japanese adult patients regularly visiting Shinshu University Hospital in Matsumoto, Japan, for chronic liver disease will complete a standardized questionnaire on the presence of PoPH symptoms. If the respondent has signs of possible PoPH, ultrasound echocardiography (UCG) will be performed as a primary screening. In the case that UCG findings indicate pulmonary hypertension, the patient will be referred to a cardiologist for further evaluation, whereby a definitive diagnosis PoPH can be made. PoPH prevalence and etiology will be investigated at the time of diagnosis. Afterwards, patients with PoPH will be followed for five years for determination of survival rate.

**Discussion:**

This study will reveal the prevalence, etiology, and 5-year survival rate of PoPH in Japanese patients with chronic liver disease.

**Trial registration:**

This study is being performed at Shinshu University following registration as UMIN 000042287 on October 29, 2020.

## Introduction

Portopulmonary hypertension (PoPH) is defined as pulmonary arterial hypertension (PAH) complicated with portal hypertension most often due to chronic liver disease [1, 2]. PoPH occurs in 5–15% of patients with PAH [3, 4] and is reportedly found in 2–6% of portal hypertension cases and 1–2% of cases of liver cirrhosis [3, 5]. The real-world data on PoPH in Japan are largely unknown as compared with in Europe and the United States [3, 4]. Atsukawa et al. earlier retrospectively analyzed 186 patients who simultaneously received hepatic vein and pulmonary artery catheterization to investigate the prevalence of PoPH and identified two patients (1%) with PoPH. However, the exact prevalence of PoPH remains unclear in Japanese patients with chronic liver disease, with the low prevalence of PoPH suggesting underestimation and an incomplete clinical picture of PoPH in Japan.

The pathogenesis of PoPH is uncertain. In addition to genetic predisposition [6], thromboembolism in the portal venous system [7], inflammation [8], hyperdynamic pulmonary circulation [9], and imbalance of vasoconstrictive and vasodilatory mediators due to a reduction in affected liver metabolism [10] have been associated with PoPH development. Indeed, higher rates of PoPH were reported in patients with end-stage liver disease undergoing liver transplantation, although the prevalence of PoPH was not influenced by liver disease severity [4, 10]. No gender differences have been detected in PoPH prevalence [11], although female patients with autoimmune hepatitis (AIH) may have a higher risk of PoPH development [12]. Accumulating evidence suggests that the existence of chronic liver disease, including AIH, hepatitis C virus (HCV) infection, and alcoholic liver disease (ALD), are linked to PoPH onset [11]. However, it is unknown which chronic liver diseases are prominently involved in PoPH development in Japanese patients.

The clinical manifestations of PoPH are identical to those of PAH, i.e., dyspnea on exertion, atypical chest pain, elevated jugular venous pressure, leg edema, and others [1, 13], all of which are considered non-specific symptoms of PoPH. Therefore, the threshold for suspecting PoPH should be low since patients with chronic liver disease often exhibit dyspnea for a variety of reasons; a precise but simple medical history interview is needed for selecting candidate patients for ultrasonic echocardiography (UCG) and further examination. Moreover, as complicating PoPH in patients with chronic liver disease is a poor prognostic factor despite targeted therapy intervention [11], early detection and intervention is needed to improve prognosis in Japanese patients with PoPH.

The present study aims to address the following clinical research questions in Japanese patients with chronic liver disease:

- Observational study 1: What is the prevalence of PoPH in Japanese patients with chronic liver disease?
- Observational study 2: What are the etiology of liver disease and clinical pathophysiology of PoPH?
- Observational study 3: What is the 5-year survival rate of PoPH, and which factors influence overall survival?

## Materials and methods

**Fig 1:**
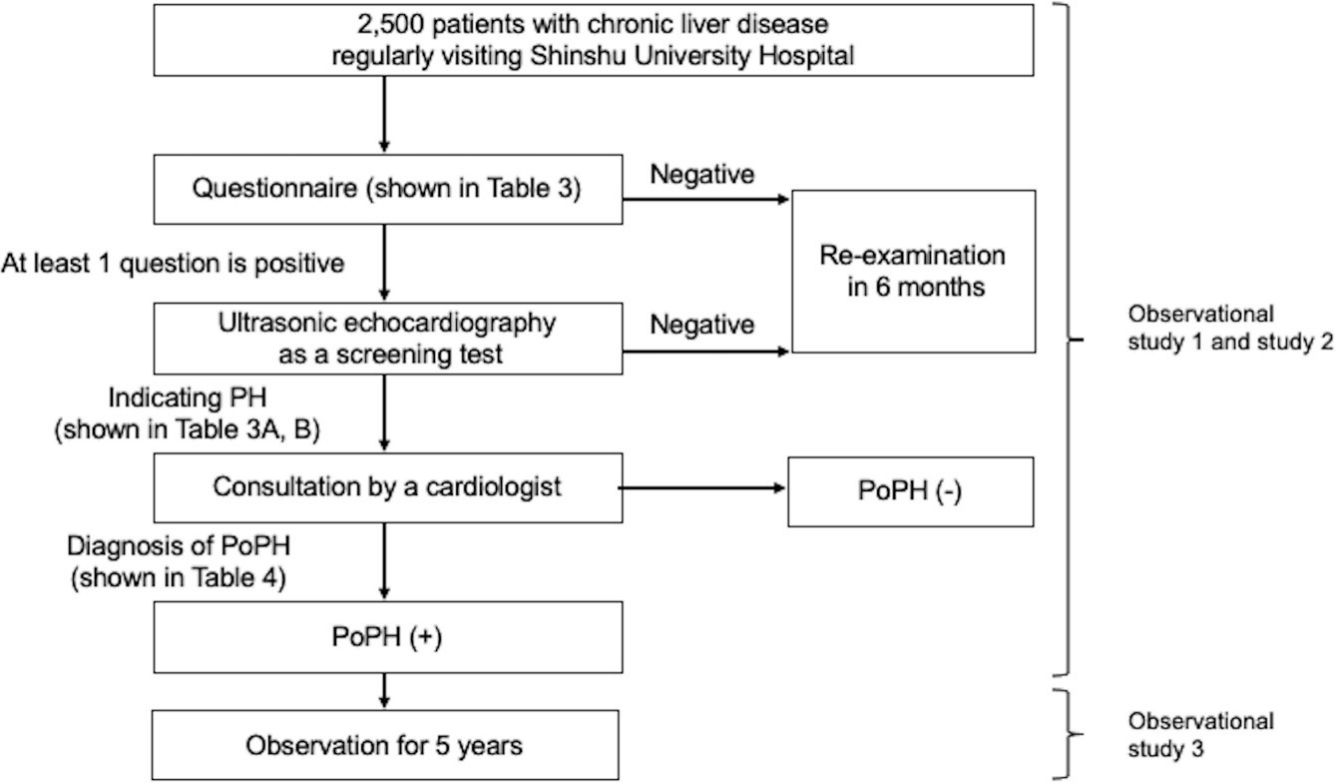
The study flowchart. Abbreviations: PH, pulmonary hypertension; PoPH, portopulmonary hypertension.

This is an ongoing, prospective, single-center, observational study that will run from October 1, 2020 to October 30, 2027. The study flowchart is presented in Fig 1. This study was reviewed and approved by the Institutional Review Board of Shinshu University School of Medicine (approval number: 4891) on September 29th, 2020. Written informed consent will be obtained from all subjects. The study is being conducted according to the principals of the Declaration of Helsinki.

### Target population

We are targeting a total of 2,500 adult patients who regularly visit Shinshu University Hospital in Matsumoto, Japan, for chronic liver disease observation or treatment.

#### Inclusion criteria

Patients in the analysis must meet all three inclusion criteria, as follows: 1) over 20 years of age, 2) having chronic liver disease, and 3) having symptoms suggestive of PoPH, as summarized in Table 1.

**Table 1:** Inclusion and exclusion criteria.

Inclusion criteria
Patients must meet all of the following items:
1. Age >20 years
2. Having chronic liver disease
3. Having any symptom indicating portopulmonary hypertension
Exclusion criteria
Patients with any of the following items are excluded:
1. Having history of chronic heart failure
2. Having history of pulmonary arterial hypertension from any other cause.
3. Unable to be followed at our hospital

#### Exclusion criteria

Patients exhibiting any of the following criteria are excluded: 1) having a history of chronic heart failure, 2) having PAH due to any other cause, and 3) unable to be followed at our hospital, as presented in Table 1.

#### Etiology of chronic liver diseases

The etiologies of the chronic liver diseases included in this study are as follows: 1) hepatitis B virus (HBV) infection defined as HBe antigen-positive asymptomatic carriers, chronic hepatitis, HBe antigen-negative inactive carriers, and HBs antigen-negative clinical remission regardless of cirrhosis status, according to the guidelines provided by the Japan Society of Hepatology [14], 2) HCV infection including patients with persistent HCV infection and with post-HCV eradication in a sustained virological response, based on the guidelines provided by the Japan Society of Hepatology [15], 3) ALD including patients with a drinking habit (ethanol >60 g/day for men, ethanol >40 g/day for women) and persistent elevation of aminotransferase and γ-GTP, according to the guidelines established by the Japanese Society of Gastroenterology [16], 4) nonalcoholic fatty liver disease (NAFLD)/nonalcoholic steatohepatitis (NASH), according to the guidelines provided by the Japanese Society of Gastroenterology and the Japan Society of Hepatology [17], and 5) autoimmune liver disease including AIH, primary biliary cholangitis (PBC), and primary sclerosing cholangitis (PSC), based on the guidelines provided by the Japan Society of Hepatology [18–20].

### Trial design and follow-up

Participant registration for the study will run from October 1, 2020 to October 30, 2022. All participants will answer the written questionnaire described in Table 2 in the outpatient clinic of Shinshu University Hospital. If at least one question is positive, informed consent will be obtained from the individual for enrollment in the study. Then, the participant will undergo ultrasound echocardiography (UCG) as a first-line screening. If the participant exhibits UCG-positive findings indicating a high probability of PoPH as shown in Tables 3 and 4, the patient will be referred to a cardiologist in the Department of Cardiology for further evaluation, whereby a definitive diagnosis will be made by right heart catherization findings as shown in Table 5. The frequency of PoPH patients will be analyzed (Observational study 1) along with the etiology of PoPH in chronic liver disease (Observational study 2).

**Table 2:** Standardized questionnaire on symptoms suggesting portopulmonary hypertension (translated from Japanese; respondents indicate “yes” or “no”).

1) Do you feel you cannot work comparably to other people of the same age and gender?
2) Do you feel you cannot move as fast as other people do?
3) Do you feel you cannot move at the same pace as others?
4) Do you need rest when climbing stairs or carrying heavy loads?
5) Do you ever experience shortness of breath?
6) Do you ever experience faintness?
7) Do you ever experience tiredness and/or persistent malaise?
8) Do you ever experience dizziness and/or vertigo?
9) Do you ever experience facial edema and/or pretibial edema?

**Table 3:** Echocardiographic probability of PAH in patients having symptoms indicative of PAH [21].

Peak tricuspid regurgitation velocity (m/s)	Presence of other echo PH signs	Echocardiographic probability of PAH
≤2.8	No	Low
≤2.8	Yes	Intermediate
2.9–3.4	No	
2.9–3.4	Yes	High
>3.4	Not required	

Abbreviations: PAH, pulmonary artery hypertension; PH, pulmonary hypertension.

**Table 4:** Echocardiographic signs suggesting PAH used to assess the probability of PAH in addition to tricuspid regurgitation velocity measurement in Table 3 [21].

A: Ventricles	B: Pulmonary artery	C: Inferior vena cava and right atrium
Right ventricle:left ventricle basal diameter ratio >1.0	Right ventricular outflow doppler acceleration time <105 msec and/or midsystolic notching	Inferior cava diameter >21 mm with decreased inspiratory collapse (<50% with a sniff or <20% with quiet inspiration)
Flattening of the interventricular septum (left ventricular eccentricity index >1.1 in systole and/or diastole)	Early diastolic pulmonary regurgitation velocity >2.2 m/sec	Right atrium area (end-systole) >18 cm^2^
	Pulmonary artery diameter >25 mm	

Abbreviations: PAH, pulmonary artery hypertension.

**Table 5:** Diagnosis criteria of PoPH [21–23].

1. Existence of PAH by right heart catheraization
a. Mean pulmonary artery pressure (mPAP) ≥ 25mmHg
b. Pulmonary artery wedge pressure (PAWP) ≤ 15mmHg
2. Existence of portal hypertension
Hepatic vein pressure gradient (HVPG) ≥ 5mmHg
3. Rule out of other etiology of PAH

Abbreviations: PoPH, portopulmonary hypertension; PAH, pulmonary artery hypertension.

When a PoPH diagnosis is made, the following clinical data will be collected: etiology of liver disease, age, gender, height, body weight, history of medication, drinking habit, smoking habit, New York Heart Association (NYHA) classification, 6-minute walking distance, FibroScan^®^ results, blood and urine data, thoracic-abdominal enhanced computed tomography findings, right heart catheter findings (mean pulmonary artery pressure, right atrial pressure, pulmonary artery wedge pressure, cardiac index, cardiac output, pulmonary vascular resistance, and mixed venous oxygen saturation), and echocardiographic findings.

Upon diagnosis, PoPH treatment will be initiated by a cardiologist based on the guidelines for pulmonary hypertension issued by the Japanese Circulation Society [22]. Patients will be evaluated every six months for blood and urine findings, abdominal ultrasonography results, and clinical symptoms. UCG will be performed on a yearly basis. The observation period will be five years from the time of patient enrollment and will end if the patient dies or becomes lost to follow-up. Ultimately, the 5-year survival rate of PoPH patients and factors associated with survival will be analyzed (Observational study 3).

#### Diagnosis of clinical stage

Liver cirrhosis will be diagnosed by histologic examination and/or characteristic clinical signs of advanced liver disease. Hepatocellular carcinoma will be determined by histologic examination, blood test, and/or imaging studies.

#### Endpoints

The primary endpoint in this study is the prevalence of PoPH in Japanese patients with chronic liver disease and its etiology (Observational study 1 and study 2). The secondary endpoint is the overall 5-year survival rate (Observational study 3).

### Safety

Since this study is observational, there are no direct risks associated with participation.

### Statistical considerations

#### Sample size estimation

As the exact prevalence of PoPH remains unknown, we planned to screen all patients regularly visiting our department for chronic liver disease observation or treatment. Approximately 4,800 patients visit our department yearly in addition to 300 new patient referrals. Symptoms indicating PoPH have been found in roughly half of patients with chronic liver disease in the outpatient questionnaire. Assuming that 20% of patients will decline informed consent, we aim to recruit 2,500 cases with chronic liver disease.

#### Statistical analysis

The prevalence of PoPH will be examined at the time of PoPH diagnosis. The clinical pathophysiology of PoPH will be evaluated by multivariate analysis using the following factors: etiology of liver disease, age, sex, height, weight, diagnosis, medication history, smoking history, drinking history, NYHA classification, 6-minute walking distance, laboratory tests, FibroScan^®^ results, the presence of cancer, right heart catheter findings (mean pulmonary artery pressure, right atrial pressure, pulmonary artery wedge pressure, and cardiac index), and echocardiographic findings. The changes in clinical symptoms, UCG findings, and hepatic function will be also be examined before and after treatment. Continuous variables will be statistically evaluated by means of the Mann–Whitney U test, while categorical variables will be analyzed using the chi-square test.

Overall survival rate will be examined using the Kaplan–Meier method. Independent factors influencing overall survival will be assessed by Cox regression analysis with stepwise methods.

## Discussion

This prospective observational study aims to clarify the clinical characteristics of PoPH in the Japanese population. Both cirrhosis and non-cirrhosis stage patients may become complicated with PoPH. Although the frequency of PoPH in patients with cirrhosis has been described, PoPH prevalence in chronic liver disease remains unknown. Unlike in cohorts from Western countries, where the proportion of ALD is high [24], viral hepatitis-related chronic liver disease is more prevalent in Japan. In the few studies on PoPH in Asia and Japan investigating disease prevalence and incidence, AIH and PBC were found to be more likely complicated with PoPH [5]. However, due to the small number of cases included in those investigations, the clinical picture of PoPH is uncertain. The present study will seek to answer these important questions.

A retrospective study from the United States showed that patients untreated for PoPH had a poor 5-year survival rate of 14%, which was associated with the delayed diagnosis of PoPH [25]. One reason could be that up to 60% of patients with PoPH were asymptomatic [1]. PoPH cases are contraindicated for liver transplantation due to high operative mortality and poor prognosis. Early intervention for PoPH is therefore essential to overcome this issue and improve prognosis. An established standardized questionnaire may be helpful in PoPH screening as a first diagnostic step.

During the past two months, this study’s questionnaire has been administered to 300 patients with chronic liver disease meeting the inclusion criteria. Interestingly, 153 patients (51.0%) described symptoms suggestive of PoPH. Among them, 36 patients providing informed consent underwent further UCG analysis. Two cases were found to have PAH, while one case was diagnosed as having PoPH. As previously reported, the prevalence of PoPH is expected to be very low. By recruiting a sufficient number of patients, however, we aim to determine the actual status of PoPH in Japan.

## Conclusions

This study will reveal the prevalence and etiology, as well as the 5-year survival rate, of PoPH in Japanese patients with chronic liver disease.

## List of abbreviations

AIH: autoimmune hepatitis
ALD: Alcoholic liver disease
HBV: Hepatitis B virus
HCV: Hepatitis C virus
NAFLD: Nonalcoholic fatty liver disease
NASH: Nonalcoholic steatohepatitis
PAH: Pulmonary artery hypertension
PBC: Primary biliary cholangitis
PoPH: Portopulmonary hypertension
PSC: Primary sclerosing cholangitis
UCG: Ultrasound cardiography
